# Chloroplast Genome Evolution in Four Montane Zingiberaceae Taxa in China

**DOI:** 10.3389/fpls.2021.774482

**Published:** 2022-01-10

**Authors:** Qian Yang, Gao-Fei Fu, Zhi-Qiang Wu, Li Li, Jian-Li Zhao, Qing-Jun Li

**Affiliations:** ^1^Yunnan Key Laboratory of Plant Reproductive Adaptation and Evolutionary Ecology, Yunnan University, Kunming, China; ^2^Laboratory of Ecology and Evolutionary Biology, School of Ecology and Environmental Science, Yunnan University, Kunming, China; ^3^Guangdong Laboratory of Lingnan Modern Agriculture, Genome Analysis Laboratory of the Ministry of Agriculture and Rural Affairs, Agricultural Genomics Institute at Shenzhen, Chinese Academy of Agricultural Sciences, Shenzhen, China

**Keywords:** adaptive evolution, chloroplast genome, gene loss, genomic variation, Zingiberaceae

## Abstract

Chloroplasts are critical to plant survival and adaptive evolution. The comparison of chloroplast genomes could provide insight into the adaptive evolution of closely related species. To identify potential adaptive evolution in the chloroplast genomes of four montane Zingiberaceae taxa (*Cautleya*, *Roscoea*, *Rhynchanthus*, and *Pommereschea*) that inhabit distinct habitats in the mountains of Yunnan, China, the nucleotide sequences of 13 complete chloroplast genomes, including five newly sequenced species, were characterized and compared. The five newly sequenced chloroplast genomes (162,878–163,831 bp) possessed typical quadripartite structures, which included a large single copy (LSC) region, a small single copy (SSC) region, and a pair of inverted repeat regions (IRa and IRb), and even though the structure was highly conserved among the 13 taxa, one of the *rps19* genes was absent in *Cautleya*, possibly due to expansion of the LSC region. Positive selection of *rpoA* and *ycf2* suggests that these montane species have experienced adaptive evolution to habitats with different sunlight intensities and that adaptation related to the chloroplast genome has played an important role in the evolution of Zingiberaceae taxa.

## Introduction

Even though the chloroplast genome is typically far smaller than most plant nuclear genomes, chloroplasts play a crucial role in plant survival, adaptation, and evolution (Wicke et al., 2011; Gao et al., 2019; Zhao C. et al., 2019; Dopp et al., 2021). In angiosperms, chloroplast genomes typically exhibit a conserved quadripartite structure, which includes two inverted repeat regions (IRs), a small single copy (SSC) region, and a large single copy (LSC) region (Shinozaki et al., 1986), as well as a relatively conserved set of genes, which can be categorized according to their involvement in photosynthesis, transcription, translation, and biosynthesis (Sassenrath-Cole, 1998). Chloroplast genes, usually 110–130, include two sets of four ribosomal RNA genes and 30 tRNA genes, which are capable of interacting with all mRNA codons by wiggle (Rogalski et al., 2008; Sibah et al., 2012). The stable genetic structure of chloroplast genomes facilitates a low mutation rate during evolution, which is uniparental inheritance (most angiosperms are maternally inherited), guaranteeing the stability of the chloroplast genome during evolution. Therefore, the chloroplast genome provides an ideal system for investigating species and genomic evolution (Dong et al., 2013).

The gene content of chloroplast genomes can change to facilitate the adaptation of species to specific habitats or life strategies. For example, the absence of the *ndh* gene and one of the IR regions in the chloroplast genome of *Cassytha* (Lauraceae) taxa and the absence of almost all photosynthesis-related genes in *Aeginetia indica* (Orobanchaceae) are associated with parasitic lifestyles (Song et al., 2017; Chen et al., 2020), and many chloroplasts are absent from the chloroplast genome of *Gastrodia elata* (Orchidaceae), which is mycoheterotrophic and does not rely on photosynthesis, thereby resulting in a relatively small chloroplast genome (35,326 bp; Yuan et al., 2018). These extreme examples suggest that changes in chloroplast gene content are closely associated with plant adaptation. The gene content, number, and structure of most autotrophic land plant chloroplast genomes are much more conserved. The main manifestation is that some special genes have been subjected to different selection pressures during adaptive evolution. For example, positive selection has been reported to play an important role in driving the functional diversification of *CHS* genes during the speciation of *Quercus* (Fagaceae; Yang et al., 2016). However, the adaptive evolution of most angiosperm groups, especially the Zingiberaceae, remains largely unknown.

Variation in chloroplast genomes provides plentiful and specific markers that can be used to resolve phylogenetic relationships at various levels (Wu and Ge, 2012; Li et al., 2019; Zhang R. et al., 2020). Moreover, as chloroplasts are maternally inherited in most angiosperms (Corriveau and Coleman, 1988), its conflict with nuclear phylogenetic relationships can provide insight into speciation processes, such as hybridization and incomplete lineage sorting (Degnan and Rosenberg, 2009; Joly et al., 2009; Petit and Excoffier, 2009). Thus, the comparative analysis of chloroplast genomes can be used to explore the evolution of plants.

Members of the Zingiberaceae are pantropically distributed (Wu and Larsen, 2001; Kress et al., 2002), and the family includes the genera *Cautleya*, *Roscoea*, *Rhynchanthus*, and *Pommereschea*, which are distributed in the mountains of southern Asia. The origin and evolution of these four genera have been linked to the orogeny caused by the collision of the Indian and Eurasian plates (Zhao et al., 2016), and phylogenetic reconstruction, using both chloroplast and nuclear markers, suggests that *Cautleya* and *Roscoea* are sister genera, as are *Rhynchanthus* and *Pommereschea* (Kress et al., 2002). Furthermore, field studies have revealed that *Cautleya* and *Rhynchanthus* taxa are epiphytic on rocks or tree trunks and inhabit shaded forest understories, whereas *Roscoea* and *Pommereschea* taxa are terrestrial and inhabit higher-altitude open habitats on the ground. In terms of morphology, the epiphytic genera (*Cautleya* and *Rhynchanthus*) are taller than the terrestrial genera (*Roscoea* and *Pommereschea*; Wu and Larsen, 2001; Kress et al., 2002). However, no studies have investigated the adaptive evolution of these genera. Previous studies have suggested that several chloroplast genes in *Zingiber* and *Curcuma* of Zingiberaceae, such as *clpP*, *ycf1*, *ycf2*, *psbA*, *psbD*, *petA*, and *rbcL*, are related to adaptative evolution (Gui et al., 2020; Li et al., 2020).

This study aimed to investigate the hypothesis that two pairs of sister genera (*Cautleya* vs. *Roscoea* and *Rhynchanthus* vs. *Pommereschea*) have common chloroplast genes associated with adaptive divergence to contrast habitats. Therefore, 13 newly sequenced and previously reported chloroplast genomes from *Cautleya*, *Pommereschea*, *Rhynchanthus*, *Hedychium*, and *Roscoea* taxa were collected to (1) analyze the characteristics and genes associated with adaptive evolution of these four montane genera, (2) reconstruct a chloroplast genome-based phylogeny of the Zingiberaceae and compare it with a nuclear marker-based phylogenetic reconstruction, and (3) explore possible adaptive evolution of these four montane genera based on associated chloroplast genes and phylogenies.

## Materials and Methods

### Sample Collection and Chloroplast Genome Assembly

Fresh leaves were collected from *Cautleya gracilis* (99.70°E, 24.18°N), *Rhynchanthus beesianus* (99.50°E, 22.48°N), *Pommereschea lackneri* (101.23°E, 21.99°N), *Hedychium coronarium* (planted variety, 102.72°E, 25.05°N), and *H. villosum* (101.23°E, 21.99°N) in Yunnan, China, and 45G sequence data were generated for each species using the Illumina Hiseq 2500 platform (San Diego, CA, United States). A total of 277,483,161, 691,955,913, 631,731,352, 309,816,484, and 309,816,484 reads were generated for *C. gracilis, R. beesianus, P. lackneri, H. coronarium*, and *H. villosum*, respectively. GetOrganelle was used to execute the *de novo* assembly of the five chloroplast genomes (− R 15 − k 105,121; Jin et al., 2020), and several previously reported chloroplast genomes from the members of the Zingiberaceae were used as references for automatic annotation and manual adjustment, which were performed using GeSeq and DOGMA, respectively (Wyman et al., 2004; Michael et al., 2017). To ensure accuracy, the coding sequences were further confirmed by online BLAST searches in NCBI. Finally, a circular map of each annotated complete chloroplast genome was drawn using Organellar Genome DRAW (Lohse et al., 2007).

### Genome Structure and Sequence Variation Analysis

A total of 13 representative chloroplast genomes, including the five newly sequenced ones, were aligned using the Mauve plugin (Darling et al., 2004) in Geneious R8 (Biomatters Ltd., Auckland, New Zealand), with the default parameters to detect inversions and rearrangements. As the chloroplast genome borders of different species typically exhibit varying degrees of contraction and expansion, SC/IR boundary maps and sequence differences were plotted according to the length differences of the four regions and the distribution of related genes.

Even though chloroplast genomes are relatively conserved, structural differences and internal mutations exist between species. To determine the sequence variation of protein-coding genes, we aimed to identify potential DNA barcode genes that may be available in the future. Protein-coding sequences were aligned using MAFFT version 7.308 (Standley, 2013), and genome divergence and variation hotspots were identified using mVISTA (Frazer et al., 2004). Finally, nucleotide diversity (π) was calculated through sliding window analysis using DnaSP version 5 (Librado and Rozas, 2009), with a window length of 600 bp and step size of 50 bp.

### Molecular Evolution Analysis

Mean amino acid usage frequency was mapped using Circos version 0.69 (Krzywinski et al., 2009), and amino acids were calculated using Geneious R8 (Biomatters Ltd.). To calculate rates of synonymous (Ks) and non-synonymous (Ka) substitution and their ratio (Ka/Ks), the nucleotide sequences of protein-coding genes shared among the four species (*C. gracilis*, *R. tibetica*, *P. lackneri*, and *R. beesianus*) were extracted and aligned separately using MAFFT version 7.308. Before calculation, gaps and stop codons between the compared sequences were removed. As the YN model considers sequence evolution characteristics (e.g., transition/transversion ratio and codon usage frequency), it has been used increasingly in molecular evolution research (Yang and Nielsen, 2000; Zeng et al., 2017; Zhang R. T. et al., 2020). Thus, the YN algorithm was chosen in KaKs_calculator (Zhang et al., 2006) to illustrate the Ka/Ks value and perform selective pressure analysis. Genes with evidence of positive selection (Ka/Ks > 1) along each branch were identified using the improved branch-site model in PAML (Yang, 2007). The targeted branch(es) was assigned as the foreground branch and the remains served as background branches (Zhang et al., 2005). Finally, a likelihood ratio test (LRT) was used to compare a model (model = 2, NSsites = 2, omega = 1, fix_omega = 0) of positive selection on the foreground branch with a null model (model = 2, NSsites = 2, omega = 1, fix_omega = 1), where no positive selection occurred on the foreground branch. The LTR and corresponding *P* values were calculated using the chi-squared module in PAML.

Previous studies have suggested that chloroplast RNA editing can improve transcript stability, contribute to the regulation of chloroplast gene expression, and enable genes to produce multiple protein products, thereby expanding the original genetic information (Hanson et al., 1996). To investigate the role of RNA editing mechanisms in the evolution of the Zingiberaceae, PREP-cp (Mower, 2009) was used to predict RNA editing sites, with a parameter threshold (cutoff value) of 0.8 to ensure prediction accuracy.

### Phylogenetic Analysis

The Zingiberaceae phylogeny was reconstructed using the chloroplast genome (whole genome or protein-coding only) and internal transcribed spacer (ITS) sequences. In addition to the five newly sequenced chloroplast genomes, other chloroplast genomes and all ITS sequences were downloaded from the NCBI database (Supplementary Table 1). In total, 47 chloroplast genomes and 54 ITS sequences, which each represented 20 genera were selected and aligned using MAFFT. Sequences from species in the Costaceae and Musaceae were also obtained for use as ingroups and outgroups, respectively. Modeltest version 3.7 (Posada and Crandall, 1998) was used to determine the best fitting model, based on Akaike Information Criterion (AIC) score (David and Buckley, 2004). Maximum-likelihood (ML) phylogenetic analysis was conducted using RAxML version 8 (Alexandros, 2014), with 1,000 bootstrap replicates, and Bayesian inference (BI) analysis was performed using the Markov Chain Monte Carlo (MCMC) algorithm in MrBayes version 3.2 (Ronquist and Huelsenbeck, 2003), with 1,000,000 generations and sampling once every 1,000 generations. The first 25% of trees from all runs were discarded as burn-in, and the remaining trees were used to construct a majority-rule consensus tree.

## Results

### Chloroplast Genome Characterization and Structure

**FIGURE 1:**
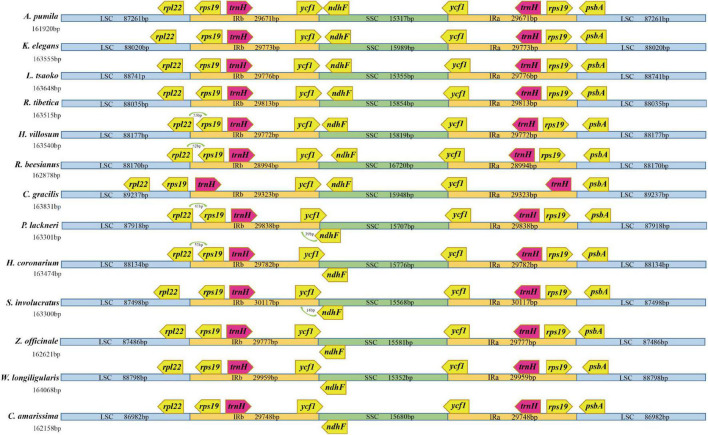
Comparison of chloroplast genome structure in Zingiberaceae. IR (inverted repeat), LSC (large single copy) and SSC (small single copy) regions and border genes are indicated.

The five newly sequenced chloroplast genomes (162,878–163,831 bp, 36.0–36.1% GC content) possessed the typical quadripartite structure, including an LSC region (87,918–89,237 bp, 33.8–33.9% GC content), SSC region (15,707–16,720 bp, 29.3–29.6% GC content), and a pair of IR regions (IRa and IRb; 28,994–29838 bp, 41.0–41.4% GC content). Except for *C. gracilis*, which was missing the *rps19* gene, the chloroplast genomes contained 133 genes, including 87 protein-coding genes, eight ribosomal RNA genes, and 38 tRNA genes (Supplementary Table 2). Of the 133 genes, 15 (*atpF*, *petB*, *petD*, *ndhA*, *ndhB*, *rpoC1*, *rps16*, *rpl2*, *rpl16*, *trnA-UGC*, *trnI-GAU*, *trnV-UAC*, *trnL-UAA*, *trnG-UCC*, and *trnK-UUU*) contained a single intron and 3 (*rps12*, *clpP*, and *ycf3*) contained two introns. The annotated complete chloroplast genome sequences were deposited in NCBI (GenBank accession numbers: MW769779–MW769783). Meanwhile, the lengths and GC contents of chloroplast genomes from all Zingiberaceae taxa (13 species and 12 genera) ranged from 161,920 bp (*Alpinia pumila*) to 164,068 bp (*Wurfbainia longiligularis*; Figure 1 and Supplementary Figure 1) and from 36.0 to 36.2%, respectively. More specifically, the lengths and GC contents of the LSC regions ranged from 86,982 bp (*Curcuma amarissima*) to 89,237 bp (*C. gracilis*) and from 33.7 to 34.0%, whereas those of the SSC regions ranged from 15,317 bp (*A. pumila*) to 16,720 bp (*R. beesianus*) and from 29.2 to 30.0%, and those of the IR regions ranged from 28,994 bp (*R. beesianus*) to 30,117 bp (*Stahlianthus involucratus*) and from 40.9 to 41.4% (Supplementary Table 3).

Moreover, variation at the SC-IR boundary and contraction and expansion were observed (Figure 1). The *rpl22* and *rps19* were located at the LSC-IRb junction, and *ycf1* and *ndhF* were located at the SSC-IRb junction. In *R. beesianus*, *P. lackneri, H. villosum*, and *H. coronarium*, the *rpl22* gene crossed the LSC-IRb boundary, with 52, 41, 53, and 53 bp located in the IRb region, respectively. Interestingly, in *C. gracilis*, the *rps19* gene, which was represented by a copy in both the IRa and IRb regions of the other genomes, was only represented by a single copy in the LSC region. In *P. lackneri* and *S. involucratus*, the *ndhF* gene in crossed the SSC-IRb boundary, with 39 and 14 bp in the IRb region, respectively, the *ycf1* gene crossed the SSC-IRa boundary in all 13 chloroplast genomes, with variable sequence lengths in the SSC region. The IRa-LSC boundary was relatively stable, except that the *C. gracilis* genome lacked an *rps19* gene (Figure 1). No gene rearrangements or inversions were observed (Supplementary Figure 2).

**FIGURE 2:**
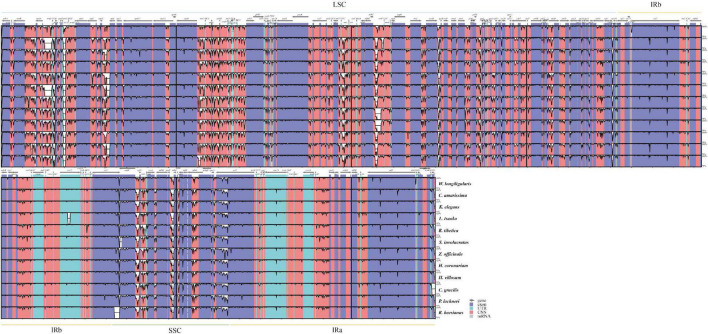
Variation level of the Zingiberaceae chloroplast genome sequences, the y-axis indicates the level of variation (between 50 and 100%) and the x-axis represents the coordinate in the chloroplast genome.

Sequence mutates indicated that the chloroplast genomes of Zingiberaceae taxa were highly conserved (Figure 2). The coding regions were more conserved than the non-coding regions, and the IR regions were less variable than the single-copy regions. Four protein-coding regions (*psbM*, *rps12*, *rpl22*, and *ycf1*), which possessed > 25% variability (Supplementary Figure 3) could be used for DNA barcode research in the future.

### Selection and Evolution of the Protein-Coding Genes

Leucine (10.3%), isoleucine (8.8%), and serine (7.9%) were the most frequently used amino acids, whereas cysteine (1.1%) and tryptophan (1.7%) were the least frequently used amino acids (Supplementary Figure 4 and Supplementary Table 4). The nucleotide diversity of the four montane taxa was ∼0.01 (Supplementary Figure 5).

**FIGURE 3:**
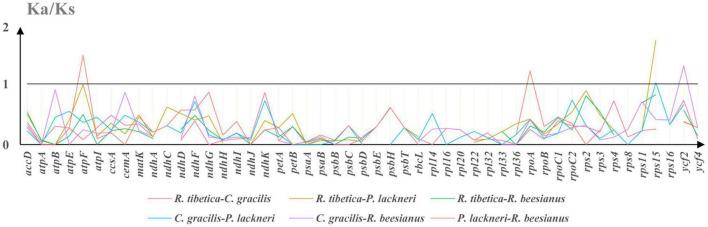
The Ka/Ks ratio of protein-coding genes of four species chloroplast genomes, and Ka/Ks > 1 suggests positive selection.

As some genes yielded Ks values of 0, which resulted in invalid Ka/Ks ratios, only 49 genes were included in the Ka/Ks analysis. KaKs_calculator suggested that four genes (*atpF*, *rpoA*, *rps15*, and *ycf2*) possessed Ka/Ks ratios of > 1 in at least one pairwise comparison among the four montane taxa (Figure 3). The genes *atpF* and *rpoA* were detected in *P. lackneri* and *R. beesianus*, respectively, whereas *rps15* was detected in *R. tibetica* and *P. lackneri*. The gene *ycf2* was detected in *C. gracilis* and *R. beesianus*. Further verification of the branch-site model revealed that the *P*-values of the targeted branches (*rpoA* and *ycf2*) were significant and retrieved sites under positive selection using the Bayes Empirical Bayes (BEB) method (Supplementary Table 5).

A total of 76–81 RNA editing sites were predicted in 25–27 genes (Supplementary Table 6). The *ndhB* gene contained the most predicted editing sites (9–11), which is consistent with findings in other plants, such as rice, maize, and tomato (Freyer et al., 1995). Meanwhile, *ndhD* contained 7–9 predicted editing sites, whereas *ndhF* contained 5–7 predicted editing sites, and the other genes contained between 0 and 7 predicted editing sites (*ndhA*, 4–7; *rpoB*, *accD*, 4–5; *ycf3*, 4; *rpoC2*, *matK*, 3–5; *rpl20*, *rpoA*, *rps14*, 3; *ndhG*, 2–3; *petB*, *rpoC1*, 2; *atpB*, *atpI*, *psbB*, *rps16*, 1–2; *atpA*, *atpF*, *ccsA*, *psbF*, *rps8*, 1; *clpP*, *rpl2*, *rps2*, 0–1). All predicted editing sites were C-to-U transitions, and most of the editing sites were predicted to greatly increase protein hydrophobicity but maintain the original function. While maintaining stability, it also provided a basis for adapting to different environments. More work is needed in this area in the future.

### Phylogenetic Relationships Analysis

**FIGURE 4:**
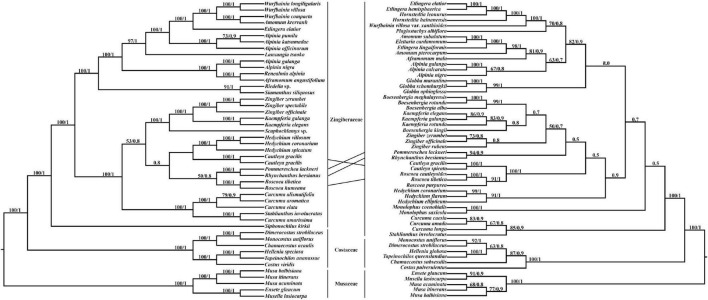
The phylogenetic tree ML (maximum likelihood) and BI (Bayesian Inference) based on 47 complete chloroplast genomes (left) and 54 ITS (internal transcribed spacer) sequences (right). Supporting values of > 50% and > 0.5 for ML and BI, respectively, were shown on the branch.

*Pommereschea*, *Rhynchanthus*, *Cautleya*, *Roscoea*, and *Hedychium* formed a monophyletic clade in the chloroplast genome tree, with BI support of 0.8, and the taxa were also closely related in the ITS tree (Figure 4). In both trees, the sister relationship of *Pommereschea* and *Rhynchanthus* was strongly supported (100% ML support and 1.0 BI support), and *Roscoea* was closely related to the *Pommereschea*-*Rhynchanthus* clade in the chloroplast genome tree, and the sister relationship of *Cautleya* and *Roscoea* was strongly supported in the ITS tree (100% ML support and 1.0 BI support).

## Discussion

In this study, chloroplast genomes from 13 species (12 genera) in the Zingiberaceae were compared to investigate the sequence structural variation and the evolution of protein-coding genes, and 47 chloroplast genomes and 54 ITS sequences were used to reconstruct phylogenetic relationships among the family. This analysis provided insight into the evolution of montane Zingiberaceae taxa.

### Loss of *rps19* Copy in *Cautleya*

Previous studies have reported that the chloroplast genomes of herbaceous plants have undergone rapid evolution, with certain structural changes, such as inversions (Doyle et al., 1992) and gene losses (Takayuki et al., 2004; Saski et al., 2005). No inversions or gene rearrangements were detected in the chloroplast genomes of the Zingiberaceae taxa included in this study. However, although most angiosperms, including most members of the Zingiberaceae, possess two copies of the *rps19* gene at the boundaries of the LSC and IR regions (Xu et al., 2015), the *Cautleya* chloroplast genome only contained a single copy of the *rps19* gene in the LSC region. Changes in *rps19* genes have been reported in several other genera, including *Dianthus* (Caryophyllaceae; Raman and Park, 2015), *Cardiocrinum* (Liliaceae; Lu et al., 2016), *Prunus* (Rosaceae; Zhao et al., 2019), and *Colobanthus* (Caryophyllaceae; Androsiuk et al., 2020). However, the changes observed in the *rps19* copies of *Cautleya* were different from those reported in other genera in two respects. First, the *rps19* copy in the IRa region of *Cautleya* was completely lost, whereas those in the IRa regions of other genera were reportedly shortened and pseudogenized. Second, the *rps19* gene in the IRb region of *Cautleya* was located in the LSC region, whereas in other taxa, the *rps19* gene remained in the IRb region.

The *rps19* protein is a component of the 40S small ribosomal subunit and is essential to both the maturation of the 3′-end of 18S rRNA and the assembly and maturation of pre-40S particles, which are related to chloroplast transcription and translation (Soulet et al., 2001; Matsson et al., 2004). The loss of *rps19* has also been observed in a few other dicot taxa (e.g., *Morus*, *Nicotiana*, *Vitis*, and *Tetrastigma*) but is relatively rare in monocots (Ravi et al., 2006; Li et al., 2015), which suggests that *rps19* is more likely to be lost or pseudogenized in dicots. The changes in *rps19* could be due to (1) partial gene duplication (Lu et al., 2016; Zhao X. et al., 2019) or (2) the contraction and expansion of IR regions (Zhao X. et al., 2019). It was suggested that there are two evolutionary mechanisms of the IR region boundary: the small amplitude amplification of the boundary gene and the recombination repair of the boundary of the LSC region. The former is an important factor for maintaining the stability of IR regions (Goulding et al., 1996). The expansion and contraction of chloroplast IR regions are relatively common (Hansen et al., 2007). Except for *Cautleya*, other Zingiberaceae taxa included in this study possessed two complete *rps19* copies, which suggests that the presence of two copies is the ancestral state within the Zingiberaceae. *Cautleya* also possesses the longest LSC region among the included taxa, which suggests that large changes in the *rps19* of *Cautleya* should be the result of LSC region expansion and repair. Previous studies have suggested that *rps19* cannot be completely removed from the IRa region through the expansion of LSC or IR regions (Raman and Park, 2015; Lu et al., 2016; Zhao X. et al., 2019; Androsiuk et al., 2020). Therefore, the complete loss of *rps19* in *Cautleya* is more likely than the suppression of *rps19* duplication by the LSC region expansion.

### Positive Selection of *rpoA* and *ycf2*

The *rpoA* and *ycf2* genes are commonly associated with positive selection, which suggests that the chloroplast genomes of *Cautleya*, *Roscoea*, *Rhynchanthus*, and *Pommereschea* have undergone adaptive evolution. Notable adaptive divergence was noted for *rpoA* in the chloroplast genomes of the sister genera *Rhynchanthus* and *Pommereschea*. The *rpoA* gene encodes the α subunit of plastid-encoded RNA polymerase, which is responsible for the expression of most genes involved in photosynthesis and is essential for chloroplast gene expression and chloroplast development (Purton and Gray, 1989; Hajdukiewicz et al., 2014; Zhang et al., 2018). The evolution of *rpoA* is complicated in angiosperms. In the Annonaceae, Passifloraceae, and Geraniaceae, *rpoA* divergence was caused by structural rearrangement and purifying selection (Blazier et al., 2016). In *Passiflora* (Passifloraceae), *rpoA* is subject to either positive or purifying selection, depending on the specific clade (Shrestha et al., 2020). In *Rehmannia* (Orobanchaceae), *rpoA* is under positive selection (Zeng et al., 2017). In this study, *Rhynchanthus*, members of which are typically epiphytic on limestone or tree trunks in forest understories at lower elevations, when compared with *Pommereschea*. Habitat differentiation, in regard to sunlight exposure, suggests that these sister genera have experienced selection based on the utilization of different light intensities.

In angiosperms, *ycf2* is the largest chloroplast gene (Huang et al., 2010) and is subjected to positive or purifying selection (Yan et al., 2019; Zhong et al., 2019). Even though previous studies have suggested that *ycf2* has been lost from the chloroplast genomes of monocots (Drescher et al., 2000; Wang et al., 2018; Mishra et al., 2019), two *ycf2* copies were present in the chloroplast genomes of the Zingiberaceae taxa included in this study. Furthermore, even though the specific function and role of *ycf2* remain unclear, studies have suggested that the gene is not essential to either photosynthesis (Drescher et al., 2000; Zhang Y. et al., 2020) or leaf patterning and is, instead, related to cell survival and possibly ATPase metabolism (Kikuchi et al., 2018; Wang et al., 2018; Zhang et al., 2018; Zhang Y. et al., 2020). The *ycf2* gene was also reported to contribute to encoding the 2-MD AAA-ATPase complex, which is a motor protein for generating ATP required for inner membrane translocation (Kikuchi et al., 2018), and to plant cell survival (Drescher et al., 2000). The positive selection of *ycf2* suggests that the gene is involved in the adaptive evolution of the montane investigated here.

### Phylogenetic Analysis

Even though the chloroplast-based Zingiberaceae phylogeny reconstruction was strongly supported and consistent with previous systematic studies (Kress et al., 2002), the phylogenetic positions of *Cautleya* and *Roscoea* in the chloroplast genome and ITS trees were inconsistent. Hybridization and incomplete lineage sorting are the most likely factors to underly phylogenetic conflict between nuclear and chloroplast genome signals (Degnan and Rosenberg, 2009; Joly et al., 2009; Petit and Excoffier, 2009). For example, *Roscoea* could be a hybrid descendant of *Cautleya* and the ancestor of *Rhynchanthus* and *Pommereschea* (Figure 4). However, incomplete lineage sorting is also possible because incomplete lineage sorting could be present at deeper-divergence lineages in angiosperms (Yang et al., 2020). Either way, this study confirmed the close phylogenetic relationships of the genera *Pommereschea*, *Rhynchanthus*, *Cautleya*, and *Roscoea*.

## Conclusion

This study reports five newly sequenced chloroplast genomes (*H. coronarium*, *H. villosum*, *C. gracilis*, *P. lackneri*, and *R. beesianus*). Even though the loss of a *rps19* gene in *Cautleya* may be associated with expansion of the LSC region and positive selection was observed for several genes in the four montane species, the functions of these genes in the adaptive evolution of this group remain unclear. Nevertheless, this study provides an important foundation for further investigation of the adaptive evolution of *Pommereschea*, *Rhynchanthus*, *Cautleya*, and *Roscoea*.

## Data Availability Statement

The original contributions presented in the study are publicly available. This data can be found here: NCBI (GenBank accessions: MW769779–MW769783).

## Author Contributions

QY, J-LZ, and Q-JL conceived and designed the study. QY, J-LZ, and LL collected and analyzed the data. QY, G-FF, Z-QW, J-LZ, and Q-JL wrote the manuscript. All authors have directly contributed to this manuscript.

## Conflict of Interest

The authors declare that the research was conducted in the absence of any commercial or financial relationships that could be construed as a potential conflict of interest.

## Publisher’s Note

All claims expressed in this article are solely those of the authors and do not necessarily represent those of their affiliated organizations, or those of the publisher, the editors and the reviewers. Any product that may be evaluated in this article, or claim that may be made by its manufacturer, is not guaranteed or endorsed by the publisher.

## Supplementary Material

Supplementary Figure 1 Gene map of the Zingiberaceae chloroplast genomes. Dashed area in the inner circle indicates the GC content of the chloroplast genome.

Supplementary Figure 2 MAUVE alignment of Zingiberaceae chloroplast genomes.

Supplementary Figure 3 Percentage of variable characters in aligned protein-coding regions of the chloroplast genomes.

Supplementary Figure 4 Average amino acid use frequency of chloroplast genomes in Zingiberaceae.

Supplementary Figure 5 Comparative analysis of nucleotide diversity in protein-coding regions of four species: **(A)** *R. tibetica*-*C. gracilis*; **(B)** *P. lackneri*-*R. beesianus*; **(C)** *R. tibetica*-*P. lackneri*; **(D)** *R. tibetica*-*P. lackneri*; **(E)** *C. gracilis*-*P. lackneri*; **(F)** *C. gracilis*-*R. beesianus*.
