# Supplementary figures and images for: Chloroplast Genome Evolution in Four Montane Zingiberaceae Taxa in China
Source: Front Plant Sci. 2022 Jan 10;12:774482. doi: 10.3389/fpls.2021.774482 (PMC8784687; doi:10.3389/fpls.2021.774482)

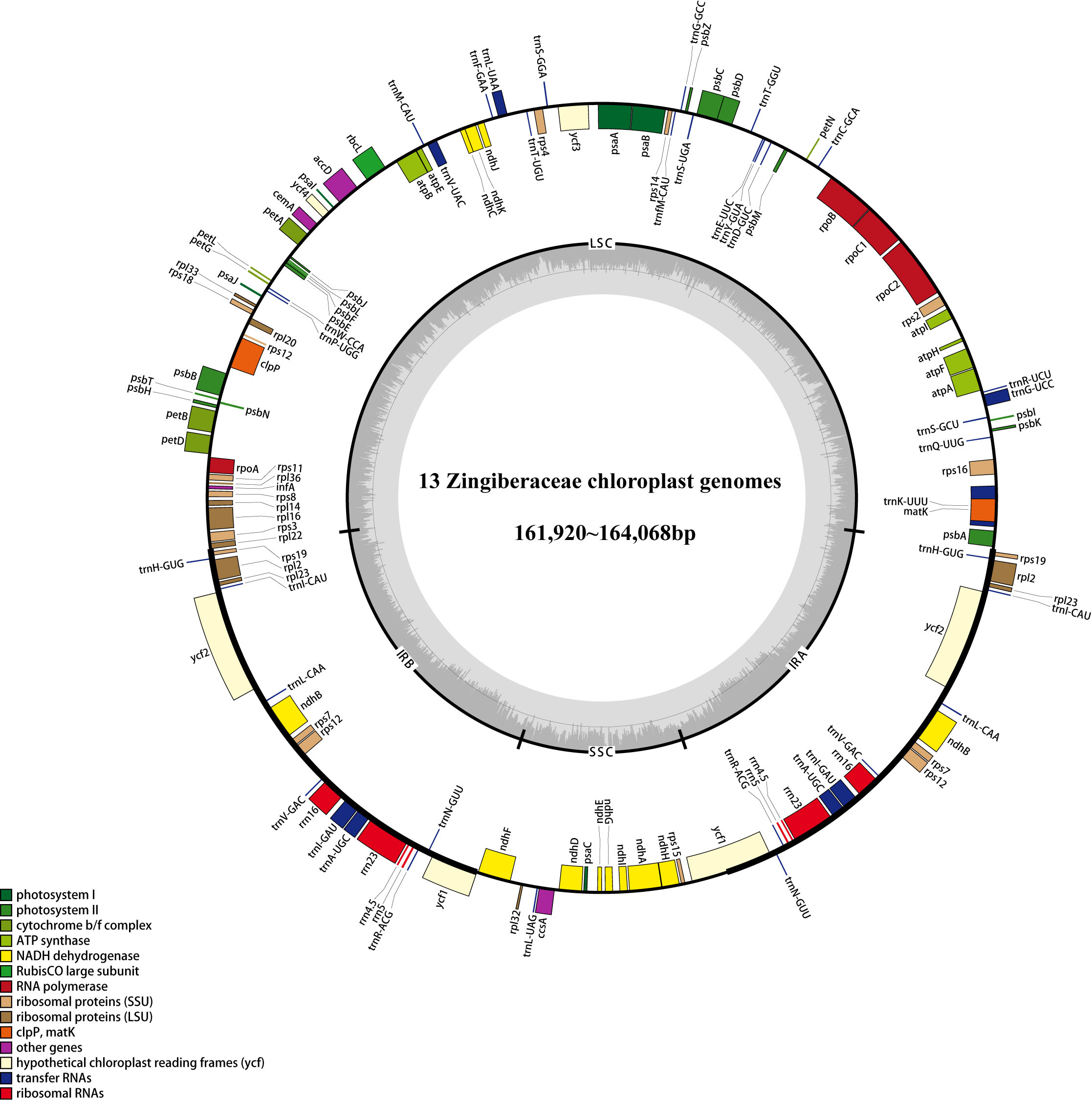

Supplement: Supplementary Figure 1 — Gene map of the Zingiberaceae chloroplast genomes. Dashed area in the inner circle indicates the GC content of the chloroplast genome. [file Image_1.JPEG]

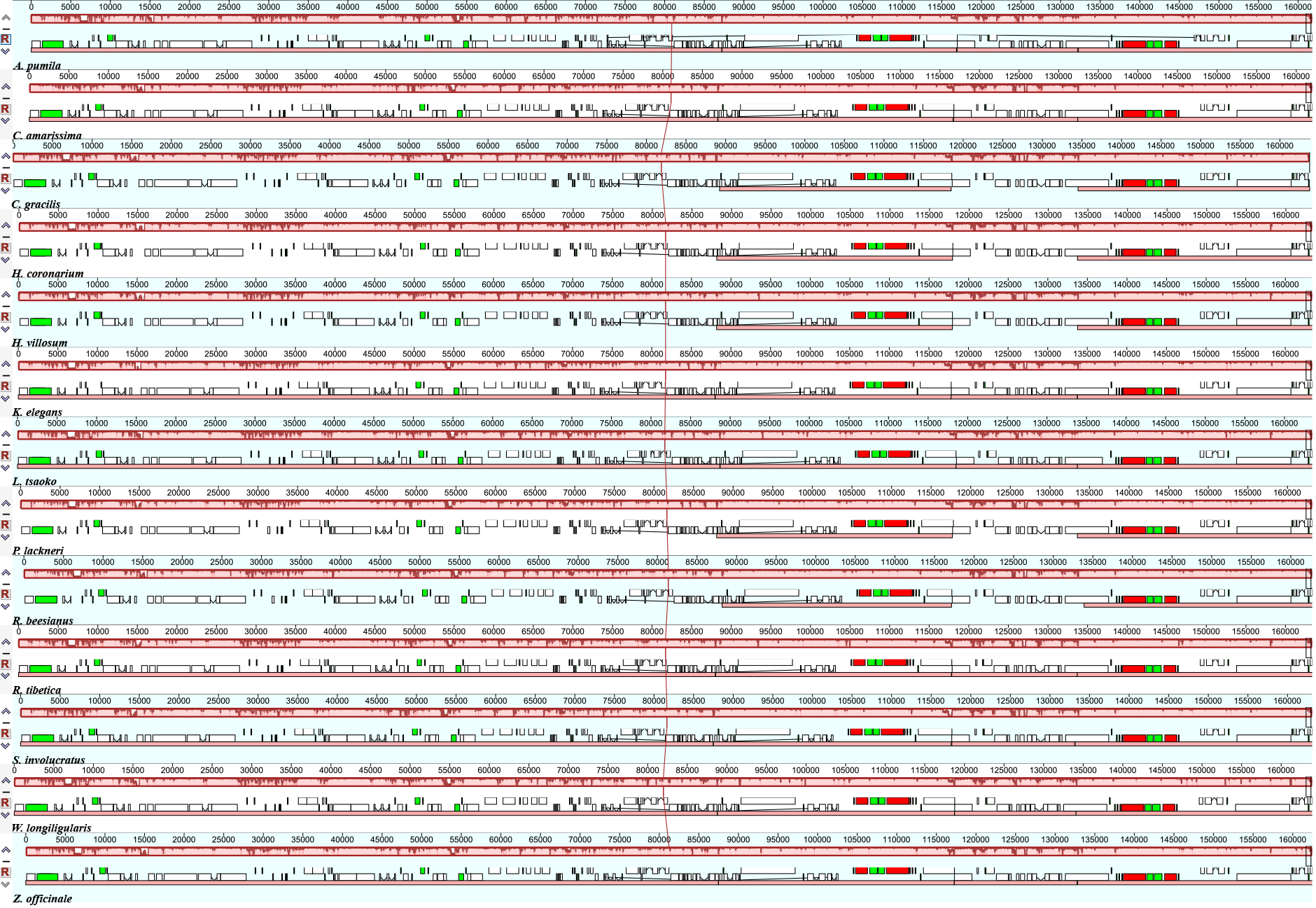

Supplement: Supplementary Figure 2 — MAUVE alignment of Zingiberaceae chloroplast genomes. [file Image_2.TIF]

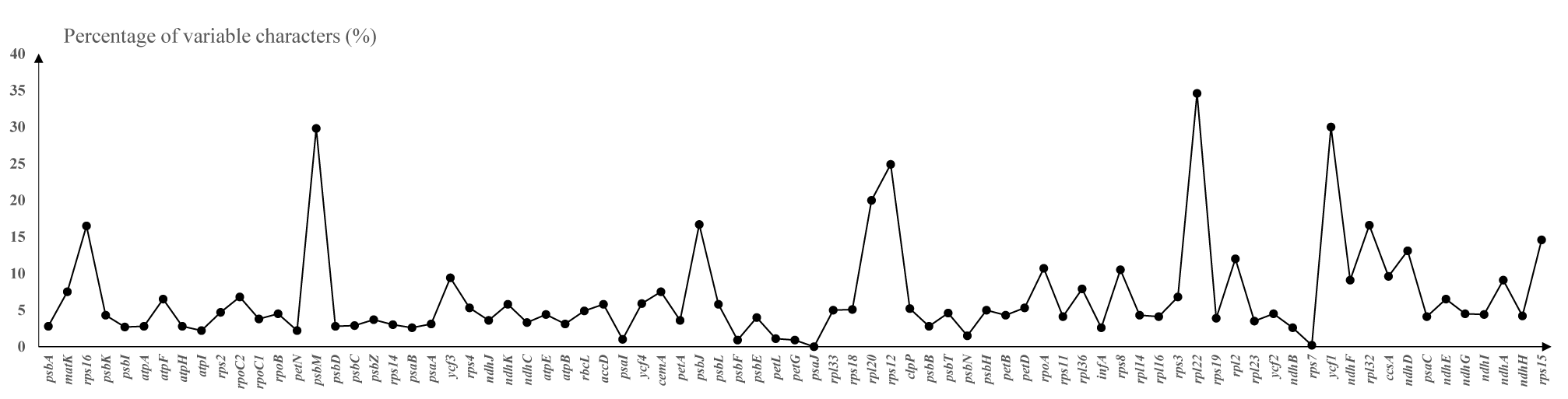

Supplement: Supplementary Figure 3 — Percentage of variable characters in aligned protein-coding regions of the chloroplast genomes. [file Image_3.TIF]

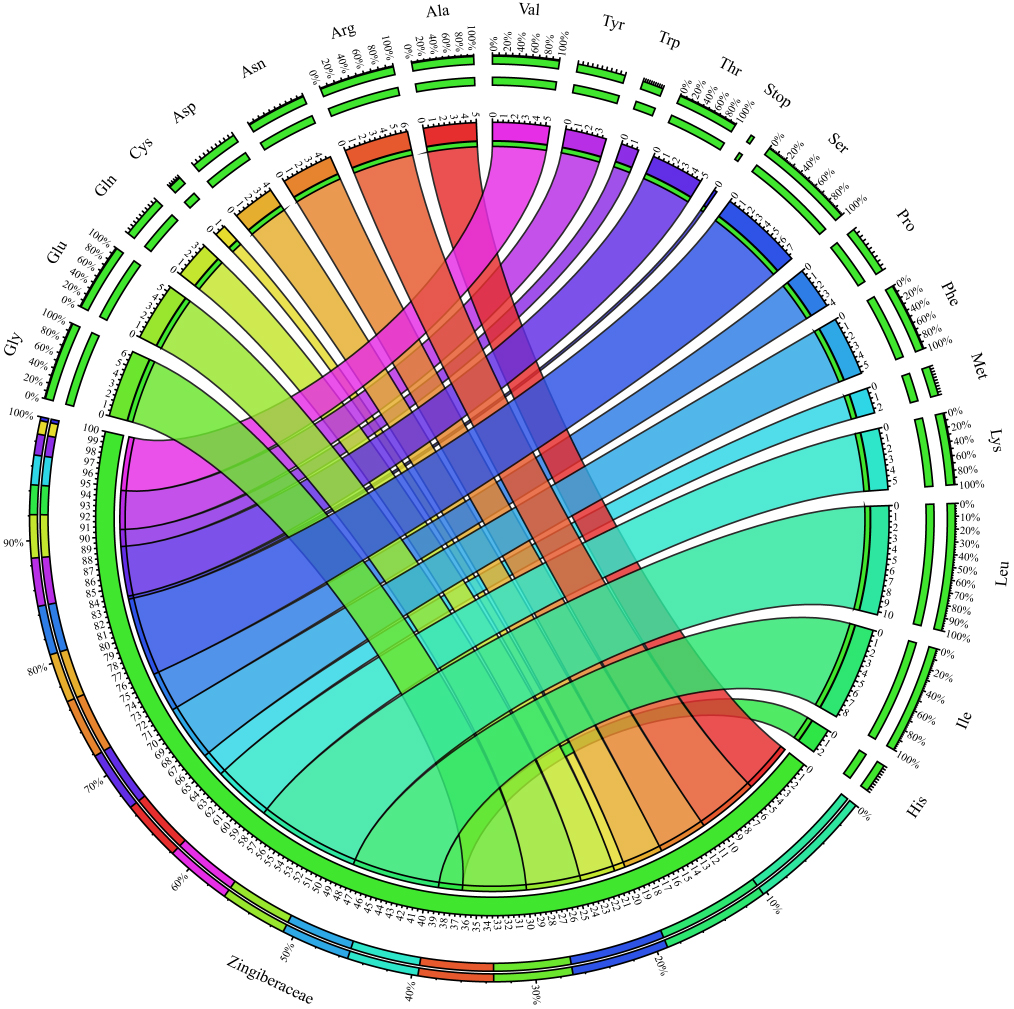

Supplement: Supplementary Figure 4 — Average amino acid use frequency of chloroplast genomes in Zingiberaceae. [file Image_4.JPEG]

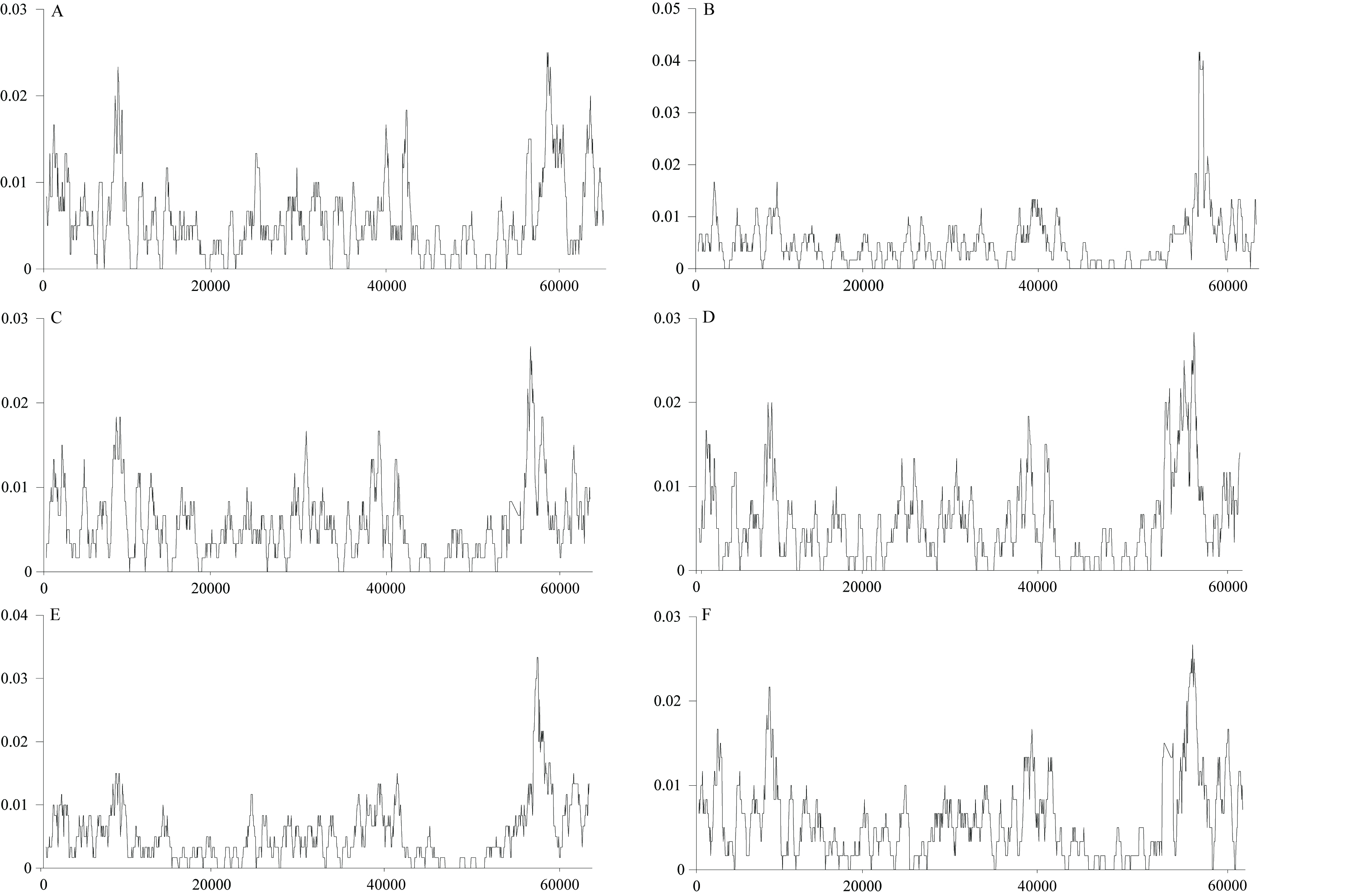

Supplement: Supplementary Figure 5 — Comparative analysis of nucleotide diversity in protein-coding regions of four species: (A) R. tibetica-C. gracilis; (B) P. lackneri-R. beesianus; (C) R. tibetica-P. lackneri; (D) R. tibetica-P. lackneri; (E) C. gracilis-P. lackneri; (F) C. gracilis-R. beesianus. [file Image_5.JPEG]
